# Feasibility and Safety of Early Oral Feeding After Radical Gastrectomy in Patients With Gastric Carcinoma: A Systematic Review

**DOI:** 10.7759/cureus.66463

**Published:** 2024-08-08

**Authors:** Wahida Ali, Wahidullah Dost, Mohammad Nazir Zaman, Mohammad Qaher Rasully, Jamaluddin Niazi, Farzad Qasemi, Raisa Dost, Wahida Dost, Danyal Bakht, Syed Faqeer Hussain Bokhari

**Affiliations:** 1 General Surgery, Jamhuriat Hospital, Kabul, AFG; 2 Medicine and Surgery, Kabul University of Medical Sciences, Kabul, AFG; 3 Cardiovascular Surgery, Punjab Institute of Cardiology, Lahore, PAK; 4 Cardiac Surgery, Kabul University of Medical Sciences, Kabul, AFG; 5 Medicine and Surgery, Mayo Hospital, Lahore, PAK; 6 Surgery, King Edward Medical University, Lahore, PAK

**Keywords:** radical gastrectomy, gastric carcinoma, review, gastrectomy, early oral feeding

## Abstract

This systematic review examines the feasibility and safety of early oral feeding (EOF) after radical gastrectomy in patients with gastric cancer. A comprehensive literature search identified eight eligible studies, including both clinical trials and cohort studies, conducted between 2011 and 2020. The review analyzed outcomes such as postoperative complications, length of hospital stay, time to first flatus/bowel movement, and changes in nutritional markers. The findings suggest that EOF is generally feasible and well-tolerated, with high adherence rates reported across studies. Most patients successfully initiated oral intake within 72 hours post-surgery without significant protocol deviations. Regarding safety, the studies reported comparable or lower rates of postoperative complications in EOF groups compared to traditional feeding protocols, though some noted non-significant increases in complications with EOF. Several studies observed potential benefits of EOF, including shorter hospital stays, earlier return of gastrointestinal function, and improved nutritional status. However, the results were mixed, with some studies finding no significant differences in these outcomes. While the review suggests EOF is a viable option for postoperative management after radical gastrectomy, it emphasizes the importance of patient-specific factors and close monitoring during implementation. The heterogeneity in study designs, EOF protocols, and outcome measures limits direct comparisons. Future large-scale randomized controlled trials are warranted to establish standardized EOF protocols and provide more robust evidence for this patient population.

## Introduction and background

Gastric carcinoma is a significant global health burden, ranking as the fifth most commonly diagnosed cancer and the third leading cause of cancer-related mortality worldwide [1]. In 2020, over one billion new cases of gastric cancer were reported, with a high incidence rate in Asian countries [2]. Radical gastrectomy, involving the surgical removal of part or all of the stomach, remains a cornerstone treatment for patients with resectable gastric cancer [3]. Traditionally, postoperative care after radical gastrectomy has involved a period of nil-per-os (NPO), usually after 72 hours postoperatively during which patients are restricted from oral intake. This approach is based on the belief that it allows adequate time for the surgical anastomosis to heal and reduces the risk of complications such as anastomotic leakage or delayed gastric emptying [4]. However, this period of NPO is associated with potential drawbacks, including prolonged ileus, impaired gut function, and an increased risk of malnutrition [5,6]. In recent years, the concept of early oral feeding (EOF) has gained increasing attention as a potential strategy to enhance postoperative recovery and improve patient outcomes. EOF involves the initiation of oral intake within the first few days after surgery, typically within 24 to 72 hours [7,8]. This approach is based on the premise that early enteral nutrition can stimulate gastrointestinal motility, promote intestinal mucosal repair, and reduce the risk of bacterial translocation and associated complications [9].

Evaluating the feasibility and safety of EOF in patients undergoing radical gastrectomy for gastric cancer is crucial for optimizing postoperative care and improving patient outcomes. Several potential benefits of EOF have been proposed, including reduced hospital stay, improved gut function, enhanced patient satisfaction, and reduced healthcare costs [10,11]. However, concerns regarding the potential risks, such as anastomotic leakage or delayed gastric emptying, necessitate a thorough examination of the available evidence. Despite the growing interest in EOF, there is a lack of consensus and clear guidelines on its implementation in the context of radical gastrectomy for gastric cancer. Previous systematic reviews and meta-analyses have explored EOF in various surgical contexts, but the evidence specific to gastric cancer patients undergoing radical gastrectomy remains limited and inconsistent [4,7]. By synthesizing the available evidence from clinical studies, this systematic review aims to provide a comprehensive understanding of the feasibility and safety of EOF in the specific context of radical gastrectomy for gastric cancer. The findings of this review may inform evidence-based clinical practice guidelines and pave the way for optimizing postoperative care and improving patient outcomes in this population.

## Review

Materials and methods

This systematic review was conducted in accordance with the Preferred Reporting Items for Systematic Reviews and Meta-Analyses (PRISMA) guidelines.

Search Strategy

A comprehensive literature search will be performed in the following electronic databases: PubMed, Hinari, Cochrane Central Register of Controlled Trials (CENTRAL), and Web of Science. The search strategy was developed in consultation with an experienced medical librarian and included a combination of relevant keywords and controlled vocabulary terms (e.g., MeSH terms for PubMed) related to "early oral feeding," "gastric cancer," "radical gastrectomy," "postoperative care," and synonymous terms. The search was tailored to each database and covered studies published from the inception of the databases until March 2024. No language restrictions were applied during the initial search. Additionally, the reference lists of relevant systematic reviews and meta-analyses identified during the initial search were manually screened to identify any potential additional studies that meet the inclusion criteria.

Eligibility Criteria

Studies were selected based on the following criteria: (1) study population: adult patients diagnosed with gastric cancer and undergoing radical gastrectomy (total or subtotal); (2) intervention: implementation of EOF; (3) comparison: studies with a control group receiving traditional postoperative feeding (delayed oral feeding or parenteral nutrition); (4) outcomes: studies reporting on at least one of the following outcomes: feasibility (e.g., adherence rates, successful initiation of oral intake), safety (postoperative complications such as anastomotic leakage, delayed gastric emptying, infections), length of hospital stay, time to first flatus or bowel movement, and patient-reported outcomes (e.g., quality of life, satisfaction) and (5) study design: randomized controlled trials (RCTs), prospective or retrospective cohort studies, and case-control studies.

Studies involving non-radical or palliative gastric surgeries were excluded. All gray literature, case reports, case series, reviews, editorials, and conference abstracts were excluded. Last, studies published in languages other than English or those lacking full-text availability were excluded to ensure accessibility and comprehensibility for the systematic review process.

Study Selection

The study selection process was conducted in two stages. First, two independent reviewers screened the titles and abstracts of all identified records to exclude irrelevant studies. Subsequently, the full texts of the remaining potentially eligible studies were obtained and evaluated against the predetermined inclusion and exclusion criteria. Any disagreements between the reviewers were resolved through discussion or consultation with a third reviewer.

Data Extraction

A standardized data extraction form was developed and piloted to ensure consistency in data collection. Two reviewers independently extracted relevant data from the included studies. The extracted information included authors, publication year, study design, country, sample size, patient age and sex, type of gastrectomy (total or subtotal), intervention details: EOF protocol (initiation time, diet progression, duration), comparison group details: traditional feeding protocol (delayed oral feeding or parenteral nutrition), and outcome measures: feasibility (adherence rates, successful initiation of oral intake), safety (postoperative complications), length of hospital stay, time to first flatus or bowel movement, and patient-reported outcomes. Any discrepancies in data extraction were resolved through discussion and consensus between the reviewers.

Results

Study Selection Process

**Figure 1:**
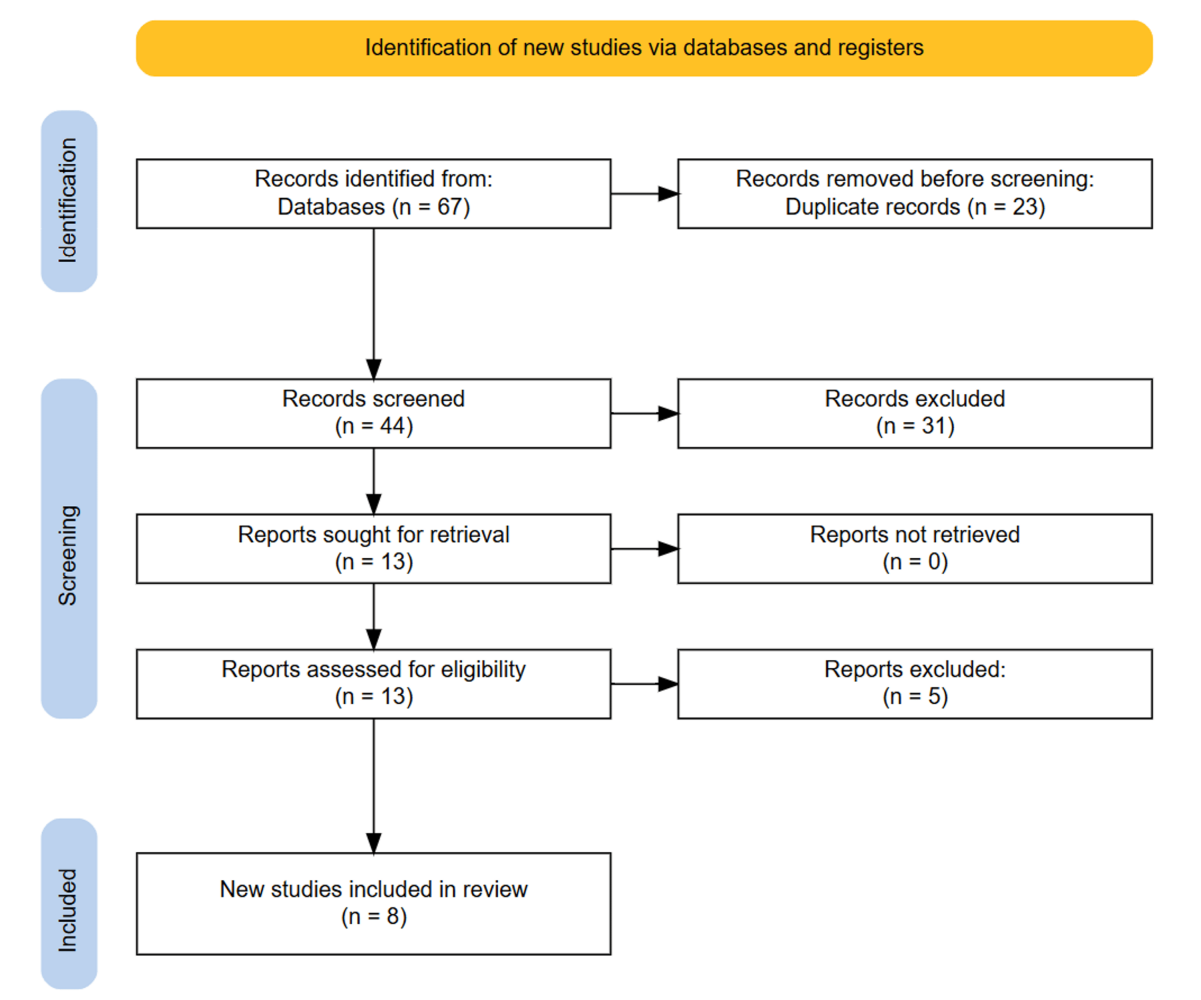
PRISMA diagram depicting the study selection process.

Following the PRISMA guidelines, the study selection process was meticulously conducted to ensure transparency. Initially, a comprehensive search across the aforementioned databases yielded 67 potentially relevant studies. After removing 23 duplicates, we had 44 unique studies. Screening the titles and abstracts of these studies resulted in the exclusion of 31 records that did not meet predefined relevance criteria. The full texts of the remaining 13 articles were then thoroughly evaluated against stringent inclusion criteria. This selection process resulted in eight studies being identified as eligible and included in the systematic review. The process is documented using a PRISMA flow diagram, detailing the number of records identified, screened, assessed for eligibility, and included in the final review (Figure 1).

Study Characteristics

Eight studies investigating the feasibility and safety of EOF in patients with gastric cancer after radical gastrectomy were included in this review. Five studies were from China and one each from Japan, Poland, and Korea. Of these eight studies, four were clinical trials and four were cohort studies, conducted between 2011 and 2020. All studies involved patients undergoing radical total gastrectomy for gastric carcinoma. Sample size varied across studies, ranging from 100 to 552 participants. In all the studies, the participants were divided into two groups: an EOF group and a control group. The EOF group received EOF after the gastrectomy procedure, while the control group followed the standard postoperative feeding protocol. The gender distribution of the participants was reported in all studies, with a higher proportion of males than females in all cases. The age of the participants was also reported in various formats. Some studies provided the mean age with standard deviation, while others reported the median age with interquartile ranges or age categories. The mean age of the participants ranged from approximately 53 to 64 years across different studies. It is important to note that the studies were conducted in different geographic regions, which may reflect variations in patient characteristics, surgical techniques, and postoperative care protocols. Additionally, the sample sizes and study designs varied, which could influence the generalizability and robustness of the findings. Overall, Table 1 provides valuable information about the study characteristics, including the sample sizes, gender distribution, and age ranges of the participants involved in these investigations of EOF after radical gastrectomy for gastric cancer. This information is crucial for understanding the context and interpreting the findings of the systematic review (Table 1).

**Table 1:** Study characteristics of included studies. EOF: early oral feeding

Author	Year	Country	Study type	Procedure	Sample size			Age (years)	
					Total	EOF group	Control group	EOF group	Control group
Lu et al. [8]	2020	China	Cohort	Radical total gastrectomy	206	n = 105 88M 17F	n = 101 86M 15F	61.69 ± 10.80 y	61.36 ± 11.72 y
Shinohara et al. [10]	2020	Japan	Cohort	Radical total gastrectomy	397	n = 223 194M 129F	n = 74 57M 17F	>80 y: 43 ≤80 y: 280	>80 y: 17 ≤80 y: 57
Jo et al. [12]	2011	Korea	Clinical trial	Radical total gastrectomy	132	n = 117 80M 37F	n = 15 10M 5F	61.1 ± 12.9 y	61.6 ± 12.5 y
Sierzega et al. [13]	2015	Poland	Cohort	Radical total gastrectomy	353	n = 185 131M 54F	n = 168 120M 48F	63 (55-72) y	64 (53-73) y
Li et al. [14]	2015	China	Clinical trial	Radical total gastrectomy	400	n = 200 104M 96F	n = 200 112M 88F	60.8 ± 5.9 y	56.0 ± 7.6 y
Wang et al. [15]	2019	China	Cohort	Radical total gastrectomy	552	n = 276 222M 54F	n = 276 221M 55F	≤60 y: 140 >60 y: 136	≤60 y: 140 >60 y: 136
Gao et al. [16]	2019	China	Clinical trial	Radical total gastrectomy	198	n = 101 68M 33F	n = 97 55M 42F	56.3 ± 10.2 y	53.9 ± 11.6 y
Wang et al. [17]	2019	China	Clinical trial	Radical total gastrectomy	100	n = 51 37M 14F	n = 49 34M 15F	53.41 ± 9.77 y	55.10 ± 8.89 y

The main findings of the included studies are summarized in the following table (Table 2).

**Table 2:** Summary of the main findings of included studies. POD: postoperative day; IV: intravenous; IQR: interquartile range; LOF: late oral feeding; E: experimental group; C: control group; EEN: early enteral nutrition; PN: parenteral nutrition; EN: enteral nutrition; PALB: prealbumin; ALB: albumin; TNM: tumor, node, metastasis (cancer staging system); EOF: early oral feeding; TOF: traditional oral feeding; DOF: delayed oral feeding; CF: control feeding

Author	Postoperative feeding type	Length of hospital stay	Postoperative complications	Tolerance to oral feeding	Changes in perioperative nutritional markers	Postoperative recovery outcomes	Other
Lu et al. [8]	EOF: day of surgery: encouraged to drink warm water; POD 1: water, small amount of clear fluid diet, and enteral nutrition; following days: gradual transition to liquid diet, semi-liquid diet, and soft food; energy supplementation: intravenous nutrition; TOF: postoperative: routine fasting; after first exhaust/defecation: gradual introduction of oral feeding; diet progression: water, clear fluid diet, liquid diet, semi-liquid diet, and soft food; energy supplementation: intravenous nutrition	EOF group: 5.85 ± 1.53 d; TOF: 7.71 ± 1.56 d (difference statistically significant)	EOF: 18 patients (17.14%); TOF: 15 patients (14.85%); incidence is higher in the EOF group but the difference is not statistically significant	EOF group: 20.95% tolerance of oral feeding; TOF group: 17.82% tolerance of oral feeding (not statistically significant)	Serum PALB: EOF: 214.52 ± 22.47 mg/L, TOF: 204.17 ± 20.62 mg/L (not statistically significant); serum ALB: EOF: 36.24 ± 5.93 g/L, TOF: 35.16 ± 4.78 g/L (not statistically significant)	First exhaust time: EOF: 2.48 ± 1.17 d; TOF: 3.37 ± 1.42 d; first defecation time: EOF: 3.83 ± 2.41 d; TOF: 5.32 ± 2.70 d (significant differences observed in mean times to first exhaust and first defecation between the two groups)	Serum levels on 5th day after surgery: gastrin: EOF: 246.30 ± 57.10 ng/L, TOF: 223.60 ± 55.70 ng/L; motilin: EOF: 424.60 ± 68.30 ng/L, TOF: 409.30 ± 61.70 ng/L (gastrin and motilin levels were statistically significantly higher on day 5 postoperatively in the early feeding group than the late feeding group)
Shinohara et al. [10]	POD 1: drink water; POD 2: thin rice gruel with liquid nutrition supplement; following days: gradual progression to regular rice porridge and solid food over 4 days, as tolerated	-	-	-	-	-	No factor was found to be significantly associated with deviation from the early oral intake regimen; however, postoperative morbidity and TNM stage showed a tendency to be associated with a deviation
Jo et al. [12]	POD 1: ingest water; POD 3 to discharge: soft diet (six times a day); first 3 PODs: IV fluid (1,400~1,500 ml/day, standard = 60 kg, 1,000 kcal)	-	Among deviating patients: 5 (33.3%); among strict adherents: 15 (12.8%) (difference statistically significant)	-	-	-	-
Sierzega et al. [13]	EOF: soft diet on POD 2 or 3; LOF: liquid diet from POD 4 to 6	EOF: 7 d (IQR 6–8), LOF: 8 d (IQR 7-15) (difference statistically significant)	Surgical complications: EOF: 27 (15%); LOF: 40 (24%) (statistically significant) General complications: EOF: 15 (8%); LOF: 38 (23%) (statistically significant) Mortality: EOF: 5; LOF: 6 (not statistically significant)	EOF: 92.97% tolerance of oral feeding; LOF: 94.64% tolerance of oral feeding (not statistically significant)	-	Time to first flatus: EOF: 2 d (IQR 1-3); LOF: 3 d (IQR 2-5) (statistically significant) Time to first defecation: EOF: 3 d (IQR 2-4); LOF: 5 d (IQR 3–7) (statistically significant)	Independent risk factors for dropout from the EOF protocol included male gender, comorbidities, and intra-operative bleeding
Li et al. [14]	E: postoperative EEN: slow infusion of warm saline (250–500 mL) through feeding tube on postoperative day 1; appropriate amount of water on the first postoperative day, followed by EN on the second day; EEN lasted for a total of seven days C: PN: total daily infusion volume of 50 mL/kg; PN lasted for a total of seven days	E: 6.8 ±1.9 d; C: 9.3 ±2.5 d (difference statistically significant)	-	-	Serum albumin (g/L): preoperative: 30.4 ± 6.7 (E); 30.5 ± 6.7 (C); POD 1: 25.3 ± 6.1 (E); 25.8 ± 5.9 (C); POD 7: 37.5 ± 7.8 (E); 31.5 ± 7.4 (C) Serum prealbumin (g/L): preoperative: 177.2 ± 23.7 (E); 174.3 ± 20.3 (C); POD 1: 136.3 ± 17.8 (E); 136.4 ± 16.8 (C); POD 7: 175.5 ± 22.6 (E); 144.5 ± 21.5 (C) (on POD 7, the levels of albumin and prealbumin in the experimental group were statistically higher compared to the control group)	Anal exhaust time (hrs): E: 67.3 ± 7.9; C: 84.6 ± 8.7 (statistically significant difference)	The incidence of fever and its duration: E: 68.7 ± 5.9 h; C: 85.4 ± 7.7 h (statistically significant difference); postoperative intestinal function recovery time: E: 69.4 ± 6.4 h; C: 86.3 ± 7.9 h (statistically significant difference)
Wang et al. [15]	EOF: POD 1: water; POD 2: clear liquid diet (glucose, sodium chloride, enteral nutrients); day 3 to discharge: liquid diet; transition: soft diet after flatus or bowel sounds appear TOF: initial feeding: water after bowel sounds or flatus; prior to feeding: nil-by-mouth with parenteral nutrition; next day: clear liquid diet; transition: soft diet after tolerating liquid diet	EOF: 6.84 ± 2.31 d; TOF: 7.72 ± 2.86 d (difference statistically significant) 4o mini	EOF: 43 patients (15.58%); TOF: 50 patients (18.12%) (incidence higher in the TOF group but the difference is not statistically significant)	EOF group: 88.41% tolerance of oral feeding; TOF group: 93.12% tolerance of oral feeding (not statistically significant)	Serum albumin (g/L): preoperative: 39.27 ± 2.34 (EOF); 39.20 ± 2.24 (TOF); POD 1: 34.33 ± 2.35 (EOF); 34.03 ± 2.84 (TOF); POD 3: 31.80 ± 3.17 (EOF); 31.78 ± 2.24 (TOF) Serum prealbumin (g/L): preoperative: 30.89 ± 2.96 (EOF); 30.86 ± 3.06 (TOF); POD 1: 38.51 ± 2.21 (EOF); 28.54 ± 2.32 (TOF); POD 3: 30.08 ± 3.64 (EOF); 30.57 ± 3.45 (TOF) (no statistical difference between nutritional markers in the EOF and TOF groups, before and after the surgery)	Time to first passage of flatus or feces: EOF: 47.19 ± 12.00 h; TOF: 58.19 ± 9.89 h (significant decrease in EOF group compared to TOF group)	-
Gao et al. [16]	EOF: oral fluid diet on POD 2; semi-liquid and soft food on 3rd day; intravenous fluids supplemented for insufficient oral intake CF: nasogastric tube inserted 30 min pre-surgery until gastrointestinal function recovery	-	EOF: Eight patients (7.92%); CF: Six patients (6.19%) (incidence higher in the EOF group but the difference is not statistically significant)	-	-	Mean time to first exhaust: EOF: 2.05 ± 0.71 d; CF: 2.50 ± 0.91 d Mean time to first defecation: EOF: 3.58 ± 0.92 d; CF: 5.17 ± 1.0 d (significant differences observed in mean times to first exhaust and first defecation between the two groups)	Serum gastrin levels (ng/L): EOF: preoperative: 198 ± 53.3; day 1: 226 ± 54.3; day 3: 244.3 ± 58.1; day 5: 248.0 ± 55.1; CF: preoperative: 197.7 ± 54.2; day 1: 218.7 ± 43.2; day 3: 217.2 ± 43.4; day 5: 221.6 ± 55.7 Serum motilin levels (ng/L): EOF: preoperative: 420.6 ± 65.6; day 1: 431.3 ± 76.3; day 3: 435.4 ± 68.1; day 5: 445.0 ± 79.4; CF: preoperative: 419.0 ± 68.1; day 1: 421.0 ± 72.3; day 3: 423.1 ± 66.1; day 5: 430.6 ± 63.8 (gastrin and motilin levels were statistically significantly higher on days 3 and 5 postoperative in the early feeding group compared to the late feeding group)
Wang et al. [17]	EOF: POD 1: encouraged to consume liquid food; POD 2 to 6: mainly liquid foods such as standard enteral nutrition (EN) liquids or milk; semi-liquids like rice soup, eggs, and soft cakes based on individual tolerances; parenteral nutrition (PN) for adequate kcal supply DOF: POD 1 to 3: limited to oral feeding; POD 4: allowed to consume liquid food; after POD 7: same oral intake as EOF group based on individual nutritional needs	EOF group: 5.18 ± 1.47 d; DOF group: 6.18 ± 2.46 d (difference statistically significant)	No significant differences in postoperative complications between the two groups	-	-	Time of first flatus (days): EOF group: 2.69 ± 0.84; DOF group: 3.12 ± 2.11 Time of first defecation (days): EOF group: 3.71 ± 1.21; DOF group: 4.24 ± 2.08 (no significant differences observed in mean times to first flatus and first defecation between the two groups)	-

Discussion

This systematic review aimed to assess the feasibility and safety of EOF after radical gastrectomy in patients with gastric cancer. The feasibility of EOF, defined as the successful initiation and adherence to an oral feeding protocol within the first 72 hours post-surgery, was consistently reported across the included studies. The reviewed studies collectively suggest that EOF is generally feasible and well-tolerated in patients undergoing radical gastrectomy for gastric cancer [8,12,13,15]. Adherence rates were high, with most patients in the EOF groups successfully initiating oral intake without significant deviations from the protocol. For instance, Sierzega et al. reported a high tolerance rate of 92.97% in the EOF group, similar to the 94.64% observed in the late oral feeding (LOF) group [13]. However, it is important to note that some studies also reported a proportion of patients deviating from or dropping out of the EOF protocol [8,12,13,16,17]. The reasons for deviations or dropouts were not consistently reported across all studies, but Sierzega et al. identified male gender, comorbidities, and intraoperative bleeding as independent risk factors for dropout from the EOF protocol [13]. This suggests that EOF is a practical and achievable strategy in the postoperative management of patients undergoing radical gastrectomy for gastric cancer.

Several factors may contribute to the feasibility of EOF in this patient population. First, the gradual progression of diet from liquids to solids, as described in the studies, likely facilitates patient tolerance and acceptance. For instance, Shinohara et al. detailed a structured progression from water to thin rice gruel and eventually to regular rice porridge and solid food over several days, which may help patients adjust more comfortably to postoperative oral intake [10]. Additionally, the support and monitoring provided by healthcare teams are crucial in ensuring successful implementation. Regular assessments of patient readiness and response to oral intake, along with appropriate adjustments to the feeding regimen, likely contribute to the high adherence rates observed. This underscores the importance of a multidisciplinary approach in managing postoperative care for these patients.

The safety of EOF was evaluated based on the incidence of postoperative complications such as anastomotic leakage, delayed gastric emptying, infections, and other related adverse events. Four studies included in this review generally reported comparable or lower rates of complications in the EOF groups compared to the control groups receiving traditional postoperative feeding protocols [8,13,16,17]. However, it is noteworthy that three studies did report a higher incidence of complications in the EOF group, although the differences were not statistically significant [8,13,16]. These findings suggest that while EOF appears to be generally safe, close monitoring and careful patient selection may be warranted to mitigate potential risks. The lower complication rates observed in some studies could also be attributed to the potential benefits of early enteral nutrition. Early oral intake may stimulate gastrointestinal motility, enhance mucosal integrity, and reduce bacterial translocation, thereby mitigating the risk of infections and other related complications. The findings of increased serum levels of motilin and gastrin in the EOF groups, as reported by Lu et al. and Gao et al., suggest improved gastrointestinal function which might contribute to these positive outcomes [8,16].

Several studies examined the impact of EOF on various postoperative recovery outcomes, including length of hospital stay, time to first flatus or bowel movement, and changes in nutritional markers. The findings were mixed, with five studies reporting significantly shorter hospital stays and earlier return of gastrointestinal function in the EOF groups compared to the control groups [8,13-16]. Sierzega et al. reported a median time to first flatus of two days in the EOF group compared to three days in the LOF group, and a median time to first defecation of three days compared to five days, respectively [13]. However, two studies did not find significant differences in these outcomes [12,17]. Regarding nutritional markers, three studies observed higher levels of serum albumin and prealbumin in the EOF groups compared to the control groups, particularly in the later postoperative days [8,14,15]. These findings suggest that EOF may contribute to improved nutritional status and faster recovery of nutritional parameters in patients undergoing radical gastrectomy for gastric cancer.

The findings of this systematic review have important implications for clinical practice and patient care. The overall feasibility and safety of EOF after radical gastrectomy for gastric cancer, as demonstrated by the included studies, suggest that this approach could be a viable option for postoperative management in this patient population. However, it is essential to consider patient-specific factors, such as comorbidities, surgical complications, and individual tolerance, when implementing EOF protocols [18,19]. Furthermore, the potential benefits of EOF, including shorter hospital stays, earlier return of gastrointestinal function, and improved nutritional status, could translate into improved patient outcomes, enhanced quality of life, and reduced healthcare costs. Early enteral nutrition has been associated with decreased rates of infectious complications, improved wound healing, and shorter lengths of hospital stay in various surgical contexts [20,21].

Nonetheless, it is crucial to acknowledge the limitations and heterogeneity of the included studies. The sample sizes varied across studies, and some studies had relatively small sample sizes, which may limit the generalizability of the findings. Additionally, the study designs, EOF protocols, and outcome measures were not consistent across all studies, making direct comparisons challenging. Future large-scale, well-designed randomized controlled trials are warranted to provide more robust evidence and establish standardized EOF protocols specific to patients undergoing radical gastrectomy for gastric cancer.

## Conclusions

This systematic review synthesizes the available evidence on the feasibility and safety of EOF after radical gastrectomy in patients with gastric carcinoma. The review was warranted due to the lack of consensus on postoperative feeding practices for this patient population. The findings suggest that EOF is generally feasible and well-tolerated (adherence rates of 88-93% reported across studies), with no significant increased risk of postoperative complications. Most patients successfully initiated oral intake within 72 hours postoperatively without significant protocol deviations. Additionally, EOF may confer potential benefits, including shorter hospital stays, an earlier return of gastrointestinal function, and improved nutritional status. However, careful patient selection and close monitoring are essential to ensure safe implementation. Future research should focus on refining EOF protocols, identifying patient-specific factors that may influence tolerance and outcomes, and conducting large-scale trials to establish evidence-based guidelines for optimal postoperative care in this patient population.
